# Rosiglitazone decreases postprandial production of acylation stimulating protein in type 2 diabetics

**DOI:** 10.1186/1743-7075-4-11

**Published:** 2007-05-09

**Authors:** Youssef Tahiri, Fredrik Karpe, Garry D Tan, Katherine Cianflone

**Affiliations:** 1 Medicine, McGill University, Montreal, H3A 1A1, Canada; 2 Centre de Recherche Hôpital Laval, Université Laval, Québec, G1V 4G5, Canada; 3 Oxford Centre for Diabetes, Endocrinology and Metabolism, Churchill Hospital Oxford, OX3 7LJ, UK

## Abstract

**Background:**

We evaluated plasma ASP and its precursor C3 in type 2 diabetic men with/without rosiglitazone (ROSI) treatment compared to healthy non-obese men. We tested (1) whether plasma ASP or C3 are altered postprandially in subcutaneous adipose tissue or forearm muscle effluent assessed by arteriovenous (A-V) differences in healthy lean men and older obese diabetic men and (2) whether treatment with ROSI changes the arteriovenous gradient of ASP and/or C3.

**Methods:**

In this ongoing placebo-controlled, crossover, double-blinded study, AV differences following a mixed meal were measured in diabetic men (n = 6) as compared to healthy men (n = 9).

**Results:**

Postprandial arterial and adipose venous TG and venous NEFA were increased in diabetics vs. controls (p < 0.05–0.0001). ROSI treatment decreased postprandial arterial TG (p < 0.001), adipose venous NEFA (p < 0.005), reduced postprandial glucose (p < 0.0001) and insulin concentrations (p < 0.006). In healthy men, there was no change in postprandial C3, but an increase in adipose venous ASP vs. arterial ASP (p < 0.02), suggesting ASP production, with no change in forearm muscle. In older, obese diabetic subjects, arterial C3 was greater than in controls (p < 0.001). Arterial C3 was greater than venous C3 (p < 0.05), an effect that was lost with ROSI treatment. In diabetics, postprandial venous ASP was greater than arterial (p < 0.05), indicating ASP production, an effect that was lost with ROSI treatment (p < 0.01).

**Conclusion:**

Increased postprandial venous production of ASP is specific for adipose tissue (absent in forearm muscle). Increased postprandial C3 and ASP in diabetic subjects is consistent with an ASP resistant state, this state is partially normalized by treatment with ROSI.

## Background

Nowadays, adipose tissue is recognized as an important endocrine organ in humans. Not only is its function of storing unlimited energy as fat a widely recognized fact, its secretory role is subject to intense research in recent years, particularly with the increasing incidence of obesity and cardiovascular disease related to lipid homeostasis disturbance [1-4]. Recent research has disclosed a group of adipokines which include leptin, acylation stimulating protein (ASP), adiponectin, tumor necrosis factor α, IL-6 and resistin, among others. Many of these factors have been shown to play a central role in adipocyte metabolism from communication with a variety of tissues to allowing the adipocyte to sense its own energy stores, to influencing energy expenditure as well as its own mass [1-4].

In the adipocytes, through the interaction of factor B, adipsin and complement component C3 (precursor to ASP), all three proteins being secreted by adipocytes in a differentiation-dependent manner [5,6], a 76 amino acid protein of 8.9 KDa is formed: C3a-des-Arg, also identified as Acylation Stimulating Protein, (ASP) [7]. At the level of the adipose tissue, ASP acts in an autocrine fashion, stimulating free fatty acid esterification to form triglycerides (TG), by stimulating diacylglycerol acyltransferase [8,9], the enzyme that regulates the last step in TG synthesis; thus stimulating TG storage in adipocytes. In addition, ASP stimulates glucose uptake through the translocation of glucose transporters Glut 1 and Glut 4 from the intracellular pool to the cell membrane. Finally, similar to insulin, ASP inhibits hormone sensitive lipase via stimulation of phosphodiesterase, thus inhibiting lipolysis in human adipocytes [5,7,10] reviews [11,12]. ASP interacts with C5L2, a G protein coupled receptor, to initiate a signalling cascade that stimulates both fatty acid esterification and glucose transport [9,13]. While no direct role for C3 in lipid metabolism has been proposed, ASP appears to be an important determinant of postprandial lipemia in both human [14] and mouse models [15-19]. In addition, previous physiological studies showed that ASP administration decreases plasma NEFA levels and enhances postprandial TG clearance in both wildtype [15] and obese [20] mice. In complement C3(-/-) ASP deficient mice, the lack of ASP results in delayed postprandial TG clearance, which is normalized acutely through ASP injection. The mice are characterized by reduced adipose tissue with a compensatory up-regulation of energy expenditure [17-19,21,22].

In humans, fasting ASP correlates with the magnitude of postprandial TG clearance independently of fasting TG [14]. Men demonstrated greater delays in TG clearance than women, reflected by increased fasting ASP. We have previously demonstrated, using venoarterial gradients across a subcutaneous adipose tissue bed, that there is a postprandial increase in adipocyte production of ASP in healthy subjects [23]. Further, study in non-obese and obese women, demonstrated that TG clearance, NEFA uptake into adipose tissue, as well as early postprandial venoarterial ASP production were all significantly greater in obese women [24]. Interestingly, in vitro studies indicate that chylomicrons and insulin increase both ASP and C3 production in human adipocytes [25-27].

Both ASP and its precursor C3 have been suggested to be altered in disease states including diabetes and cardiovascular disease, but a detailed comparison between the two proteins has not been determined (review [28]). ASP and C3 are both increased in obesity [28], even in very young children [29]. ASP and C3 levels also increase in diabetics [28], even in the absence of changes in body weight [30]. First degree relatives of type 2 diabetics have increased C3 [31] and in a population-based cohort study, C3 was associated with increased risk for diabetes development [32].

The aims of this study were to evaluate plasma ASP and its precursor C3 in type 2 diabetic men with and without treatment with rosiglitazone (ROSI) compared to healthy non-obese men. We tested (1) whether plasma ASP or C3 concentrations are altered postprandially in adipose tissue or muscle bed effluent as assessed by arterio-venous differences in healthy lean men and diabetic men and (2) whether treatment with ROSI changes the arterio-venous gradient of ASP and/or C3.

## Materials and methods

### Subjects

Nine non-obese healthy men and six diabetic men (before and after treatment with ROSI) were studied. The present diabetic subjects are a subset of a larger double-blind, placebo-controlled, cross-over study [33]. Inclusion criteria of diabetic subjects included the following: age 30–70 years, fasting plasma glucose 7–12 mmol/L and a BMI greater than 24 kg/m^2^. Subjects were excluded if treated previously with oral hypoglycaemic agents or any medication known to affect glucose metabolism. No subjects had cardiac, liver, renal, or chronic disease or microvascular complications. Subjects were administered ROSI 4 mg twice daily for 12 weeks followed by placebo for 12 weeks, or vice versa (split equally between the subjects). At the end of each period, patients were evaluated following a 10-h overnight fast, with no alcohol or vigorous exercise in the preceding 24-h. The study was approved by the clinical research ethics committee at all authors' institutions and all subjects gave their written informed consent.

### Experimental design of tissue specific arteriovenous analysis

Venoarterial (V-A) studies were conducted in a temperature-controlled room (23°C) as described in detail previously [33]. Briefly, a cannula was inserted retrogradely into a hand vein and the hand was warmed in a box at 60°–70°C so that arterialized blood samples could be obtained. A 10-cm 22-gauge catheter was then introduced over a guide wire into one of the superficial veins on the anterior abdominal wall and threaded toward the groin, so that its tip lay just superior to the inguinal ligament. Samples from this cannula represent the venous effluent from the subcutaneous abdominal adipose tissue, uncontaminated by muscle drainage and with only a minor contribution from skin. Venous blood from muscle was taken retrogradely from a vein draining the deep structures of the forearm. All catheters were kept patent with saline and heparin was not administered. The subjects rested for at least 30 min after insertion of the catheters and before any samples were taken. Subjects consumed a mixed meal containing 40 g olive oil, 400 mg emulsifier, 200 mL skimmed milk and Rice Krispies (Kellogg Company, Manchester, UK) containing 40 g fat and 40 g carbohydrate. Blood samples were taken at each time point simultaneously from all three sites for 6-h after the meal. The samples were centrifuged and plasma was stored at -70°C.

### Analyses

Plasma TG, glucose, NEFA and insulin concentrations were measured as described previously [34]. Human ASP was assayed as previously described [23,35]. The intra-assay variability was 4% whereas the inter-assay variability was 8%. Complement C3 was measured as described previously [26]. For the control group, only samples from n = 5 subjects were available for analysis. Arteriovenous (AV) differences were calculated as [venous-arterial] values. Fatty acid transcapillary flux, the net movement of fatty acid across the endothelium in the adipose tissue, was measured as: [3 × TG AV flux] + [NEFA AV flux].

### Statistics

All results are presented as means ± standard error of the mean (sem). Statistical differences for the data were calculated by 2-way repeated measures analysis of variance (RM-ANOVA). The total area under the curve (AUC) for C3 and ASP were calculated by a trapezoidal technique, with no basal subtraction. For transcapillary flux, the incremental AUC was calculated by trapezoidal technique using the fasting (t = 0) as baseline value. The groups were compared with an unpaired *t*-test or one-way ANOVA (followed by Bonferroni post hoc test) unless the data were not normally distributed, in which case a Mann-Whitney test was applied. A p value of NS indicates not significant.

## Results

### Baseline values of study subjects

Male patients with type 2 diabetes were examined before and after treatment for 12 weeks with ROSI and compared with control subjects as shown in Table 1. Subjects with type 2 diabetes were older and more obese with significantly higher circulating levels of glucose compared to control subjects. With ROSI treatment, there was a significant decrease in glucose, as expected, although values still remained higher than in control subjects. Neither fasting TG nor NEFA changed significantly.

**Table 1:** Fasting Plasma Lipid and Hormone Levels

Fasting Values	Control *n = 9*	Diabetics *n = 6*	Diabetics + ROSI *n = 6*	ANOVA
Age (years)	36.4 ± 2.9	54.3 ± 4.2**	54.3 ± 4.1**	0.002
BMI (kg/m^2^)	24.3 ± 3.0	32.2 ± 2.1**	33.2 ± 2.0**	0.007
Glucose (mmol/L)	5.3 ± 0.15	8.84 ± 0.80*	7.0 ± 0.59	0.003
Triglyceride (umol/L)	1098 ± 177	2148 ± 399**	2152 ± 535	NS
NEFA (umol/L)	513 ± 41	650 ± 99	568 ± 61	NS
Insulin (pmol/L)	70.5 ± 5.1	121.0 ± 28.6	86.7 ± 11.7	NS

Fasting plasma glucose, triglyceride, NEFA and insulin levels are presented as mean ± SEM where * p < 0.05 and ** p < 0.01 compared to control analyzed by ANOVA with Bonferroni post hoc test, where NS = not significant.

### Fasting and postprandial insulin and glucose levels in diabetics plus ROSI treatment

**Figure 1:**
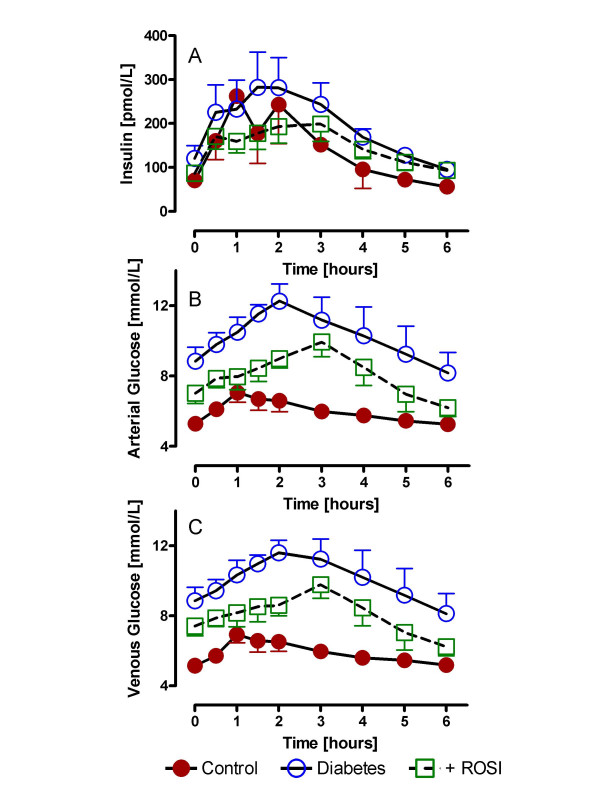
Fasting and postprandial insulin and glucose levels in diabetics plus ROSI treatment: Following a standardized mixed meal, arterial insulin (A), arterial glucose (B) and adipose tissue venous glucose (C) were measured serially from 0 to 6 hours in control (n = 9), diabetics (n = 6) and diabetics treated with rosiglitazone (ROSI). Results are expressed as average ± sem with detailed statistics presented in the text, all comparisons used RM-ANOVA.

As shown in figure 1 for all three groups, circulating insulin increased after a mixed meal. However, type 2 diabetic subjects had greater circulating insulin levels as compared to control subjects (p < 0.03 RM-ANOVA). These levels were significantly decreased with ROSI treatment (p = 0.006 RM-ANOVA) such that they were no longer significantly increased compared to control subjects (p NS). Glucose levels in the general circulation (arterial) and from subcutaneous abdominal adipose tissue drainage (venous) for the three groups of subjects are shown in Figure 1B and 1C, respectively. For both arterial and venous blood samples, glucose increased significantly postprandially, and was significantly higher in type 2 diabetic subjects as compared to control subjects (p < 0.0001 and p < 0.0001 respectively, by RM-ANOVA). While ROSI treatment significantly decreased circulating glucose (p < 0.0001 and p < 0.0001 for arterial and venous, respectively, by RM-ANOVA), glucose levels still remained significantly above controls (p < 0.0001 and p < 0.0001 for arterial and venous, respectively, by RM-ANOVA).

### ROSI effects on plasma TG and non-esterified fatty acids (NEFA)

**Figure 2:**
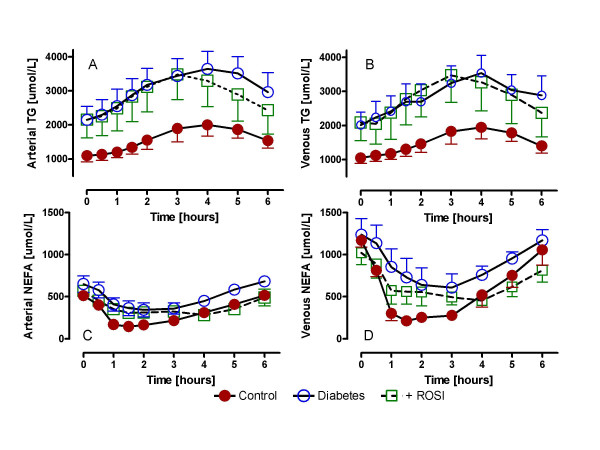
ROSI effects on plasma TG and non-esterified fatty acids (NEFA): Following a standardized mixed meal, arterial TG (A), adipose venous TG (B), arterial NEFA (C) and adipose venous NEFA (D) were measured serially from 0 to 6 hours in control (n = 9), diabetics (n = 6) and diabetics treated with rosiglitazone (ROSI). Results are expressed as average ± sem with detailed statistics presented in the text, all comparisons used RM-ANOVA.

As shown in figure 2, there were significant differences in fasting and postprandial TG and NEFA between the groups. Type 2 diabetics had markedly higher fasting and postprandial TG levels as compared to controls for both arterial (Figure 2A) and venous (Figure 2B) TG, p < 0.0001 and p < 0.005, respectively, by RM-ANOVA. While ROSI significantly reduced postprandial arterial TG in type 2 diabetics (p < 0.001, by RM-ANOVA), there was no significant change in venous TG levels (p NS, by RM-ANOVA), and in both cases, TG remained substantially greater than the control subjects (p < 0.05 and p < 0.005 for arterial and venous respectively, vs. controls, by RM-ANOVA).

Arterial and venous NEFA levels are shown in figure 2C and 2D. There was no difference in arterial NEFA levels (2C) between all three groups, although in each case there was a postprandial decrease between 1 and 3 hours. With respect to venous output, for all three study groups there was a positive (and significant) NEFA production across the adipose tissue bed (venous vs. arterial, p < 0.0001). However, there were differences in venous NEFA output between the three groups. The control group demonstrated a striking NEFA suppression while the diabetic group displayed minimal NEFA suppression of venous output, p < 0.05 diabetics vs. controls. ROSI treatment led to a normalization of the NEFA suppression (p < 0.005 diabetics vs. ROSI, and p NS for ROSI vs. control) that appeared to be maintained even longer than in the control group (up to 4–5 hours).

### Adipose tissue and skeletal muscle venoarterial ASP and C3 changes in healthy non-obese men

**Figure 3:**
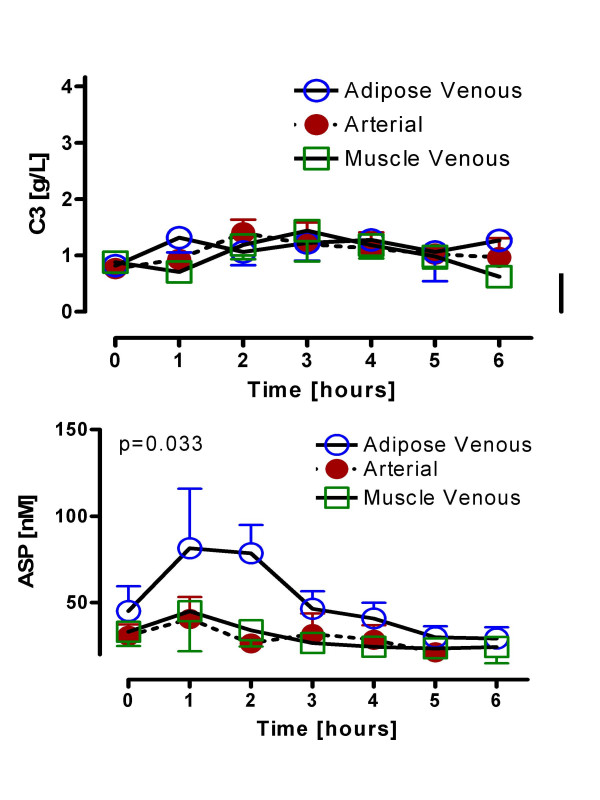
Adipose tissue and skeletal muscle venoarterial ASP and C3 changes in healthy non-obese men: Following a standardized mixed meal, complement C3 (A), and ASP (B) were measured serially from 0 to 6 hours in control (n = 9 for ASP, n = 5 for C3) subjects in arterial, adipose venous and muscle venous samples. Results are expressed as average ± sem with detailed statistics presented in the text, all comparisons used RM-ANOVA.

We first examined postprandial changes in ASP and C3 (precursor to ASP) in normal healthy men (Figure 3). For C3, samples were available only for n = 5 men. There was no postprandial change in C3 levels in control subjects for either arterial or adipose tissue venous output (Figure 3A). Similarly, there was no change in muscle bed output (Figure 3A), nor was there any significant difference between the three tissue sources. By contrast, as shown in Figure 3B, while general circulating arterial ASP concentrations remained constant postprandially, there was a marked increase in venous adipose tissue effluent postprandially, demonstrating a positive adipose tissue ASP production in men (p < 0.02). However, there was no change in ASP concentrations from the muscle venous effluent, as compared to general circulating levels.

### ROSI decreases complement C3 and ASP production

**Figure 4:**
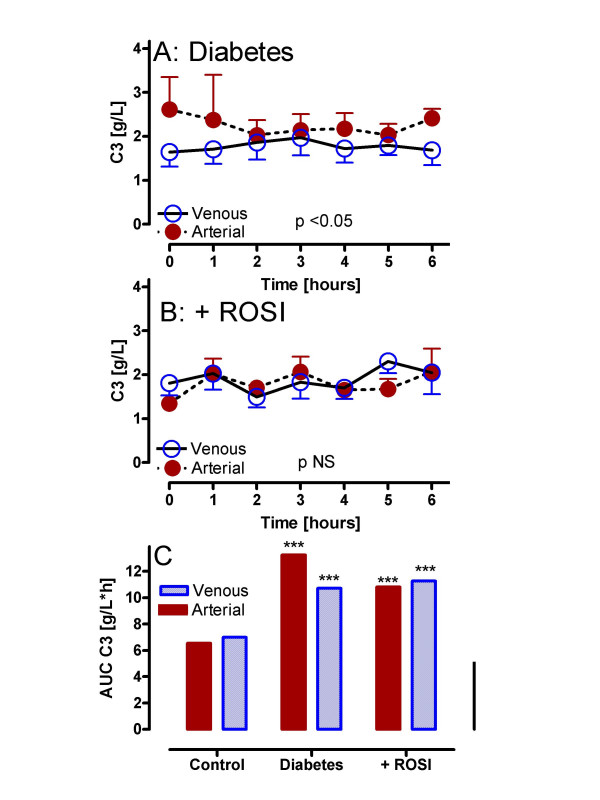
ROSI decreases complement C3 in diabetic subjects: Following a standardized mixed meal, arterial and adipose venous complement C3 were measured serially from 0 to 6 hours in diabetics (A: n = 6) and diabetics treated with rosiglitazone (B: n = 6 ROSI). Results are expressed as average ± sem where the statistical comparison of venous vs. arterial for each group is provided within the graph (using RM-ANOVA). Detailed statistics comparing groups are presented in the text. In Panel C, results are given for calculated total area-under-the-curve (AUC) for C3 in control, diabetics and diabetics treated with ROSI, where *** p < 0.001 vs. the matched arterial or adipose venous control.

Results for complement C3 in older, obese diabetic subjects pre and post treatment are shown in Figure 4. By contrast to control subjects, fasting arterial C3 was higher in diabetic subjects (2.6 ± 0.74 vs. 0.76 ± 0.06, p < 0.05). As shown in Figure 4A, postprandial arterial C3 was significantly increased relative to venous (p < 0.05). With ROSI treatment, fasting arterial C3 was decreased (1.33 ± 0.17, p NS vs. control) and there was no longer any difference between arterial and venous concentrations (p NS, Figure 4B), similar to results in control subjects. As with the control subjects, muscle output was not statistically different from arterial levels for both diabetics pre and post ROSI treatment (data not shown). A direct comparison of C3 total area-under-the-curve (AUC) for all groups is shown in Figure 4C. Postprandial circulating (arterial) and adipose drainage (venous) C3 in diabetics pre and post treatment are all significantly greater than control C3 (p < 0.001), despite significant decreases in arterial C3 AUC in diabetics with ROSI treatment (p < 0.05).

**Figure 5:**
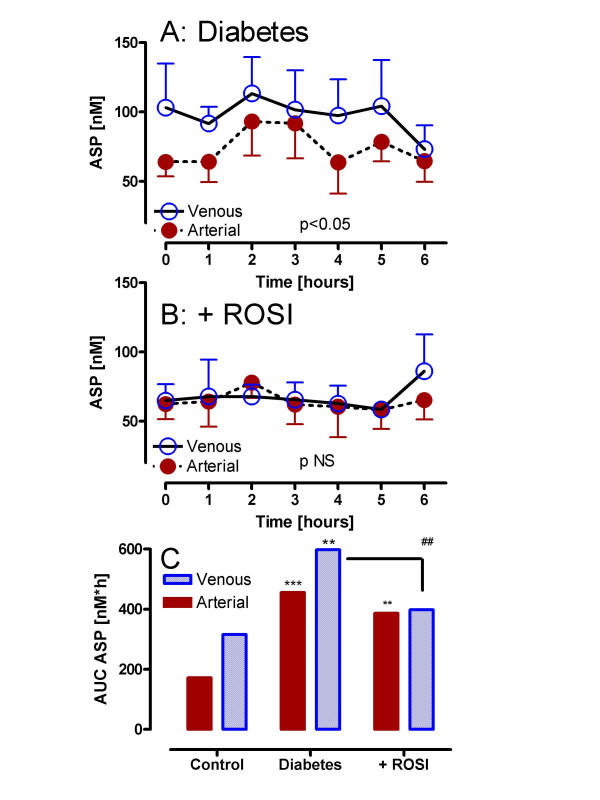
ROSI decreases ASP production in diabetic subjects: Following a standardized mixed meal, arterial and adipose venous ASP were measured serially from 0 to 6 hours in diabetics (A: n = 6) and diabetics treated with rosiglitazone (B: n = 6 ROSI). Results are expressed as average ± sem where the statistical comparison of venous vs. arterial for each group is provided within the graph (using RM-ANOVA). Detailed statistics comparing groups are presented in the text. In Panel C, results are given for calculated total area-under-the-curve (AUC) for ASP in control, diabetics and diabetics treated with ROSI, where ** p < 0.01 and *** p < 0.001 vs. the matched arterial or adipose venous control, ^## ^p < 0.01 ROSI treatment vs. untreated diabetics.

Results for ASP in older, obese diabetic subjects are shown in Figure 5. Fasting arterial ASP tended to be increased (but not significantly) in diabetic subjects (64.0 ± 10.4 diabetics vs. 38.6 ± 11.2 nM controls). Nonetheless, postprandially, there is a significant arteriovenous difference in ASP over the 6 hour period (Figure 5A, p < 0.05 by 2 way ANOVA). While there is no change in fasting circulating arterial ASP levels with ROSI treatment (62.5 ± 10.8 nM), there is no longer any significant adipose tissue generation of ASP (Figure 5B, pNS). As shown in Figure 5C, ASP total AUC in both arterial and venous pools in diabetics is significantly increased compared to control subjects (p < 0.001 and p < 0.01 respectively). With ROSI treatment, arterial ASP AUC remained significantly increased relative to control (p < 0.01), the adipose venous effluent was significantly decreased compared to pre-treatment (p < 0.01) and is no longer significantly increased relative to control venous ASP AUC (pNS).

### Fatty acid transcapillary flux

**Figure 6:**
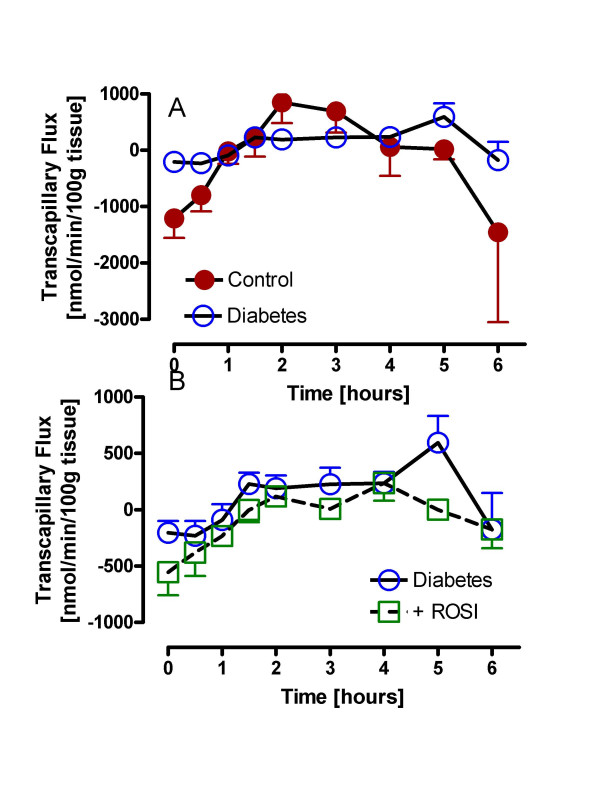
Fatty acid transcapillary flux in controls and diabetics plus ROSI treatment: Following a standardized mixed meal, TG and NEFA levels were measured and fatty acid transcapillary flux calculated as described in methods for controls and diabetics (A) and diabetics with and without treatment with rosiglitazone (ROSI)(B). Results are expressed as average ± sem with detailed statistics presented in the text.

Fatty acid transcapillary flux, which measures the net movement of fatty acids across the endothelium into adipose tissue, is shown in Figure 6 for all three groups of subjects. As shown in Figure 6A, for the controls, the flux is significantly negative at fasting (taken as baseline value), as a consequence of active intracellular adipose TG lipolysis releasing fatty acids. Conversely, during the postprandial period, the flux is significantly positive, representing a net flux of fatty acids into the adipose tissue for storage, with a robust shift from negative-to-positive-to-negative evident in the control subjects (incremental AUC 8013 ± 1828). In the diabetic subjects, both the fasting and postprandial responses are significantly blunted, with little oscillation from negative to positive (incremental AUC 2526 ± 748, ANOVA p = 0.03, p < 0.05 diabetics vs. control). With ROSI treatment (Figure 6B), there is some improvement in the negative to positive shift such that there is now no difference with the control group (incremental AUC 3233 ± 932, pNS vs. control).

## Discussion

In the postprandial period, a series of interrelated events are occurring in the adipose tissue. One of the principal functions of the adipose tissue is to store energy. This function is attained in healthy people through the interaction of a number of hormones acting on the tissue. We have previously demonstrated in non-obese and obese women that ASP is produced postprandially across an adipose tissue bed [23,24]. The present study is the first to demonstrate this in an all-male study with lean healthy men and in older, obese diabetic men, and further to demonstrate that this is particular to adipose tissue, as the ASP gradient over a muscle bed is neither positive nor negative.

Adipose tissue function is particularly disturbed in patients with type 2 diabetes, demonstrated by alterations in many adipose tissue hormones (leptin, adiponectin, ASP) both independently of, and related to, the accompanying obesity (reviews [3,36,28]). The specific focus of this study was to examine the association between postprandial NEFA and TG metabolism and ASP production in patients with type 2 diabetes compared to controls, and further to evaluate the effects of ROSI treatment. The present study demonstrated that basal C3 and ASP are increased in type II diabetics; and in spite of increased basal levels, there is further postprandial production of ASP across an adipose tissue bed. With ROSI treatment, fasting C3 decreased, yet there was little change in fasting ASP with the most striking change being a loss of postprandial ASP production.

Why are fasting and postprandial C3 and ASP increased in type 2 diabetics? Previous studies have demonstrated increased C3 and ASP in diabetic subjects (review [28]). However, to our knowledge, this is the first study examining postprandial ASP and C3 adipose tissue fluxes in diabetic subjects. Firstly, the increased BMI in these diabetic subjects partially explains the high baseline levels of ASP and C3, as obesity (without diabetes) is associated with increased ASP and C3 (review [28]). However, even in non-obese subjects, both ASP and C3 correlate with parameters of glucose and insulin homeostasis (review [28]). Even in the absence of obesity, lean diabetics have increased plasma ASP and C3 [30]. As ASP has similar effects to insulin for fat storage (stimulates TG synthesis, increases glucose uptake, inhibits lipolysis) and these patients are insulin resistant, there may initially be increased secretion of ASP to compensate for adipose insulin resistance. Further, as ASP has been shown to enhance insulin secretion in cellular and rodent models, ASP may also enhance insulin secretion in humans [37]. On the other hand, it has been shown that, in healthy men and women, fasting plasma ASP directly correlates with postprandial TG clearance [14]; delayed TG clearance is associated with higher fasting ASP, suggesting the presence of an ASP resistant state. In diabetics, as the insulin resistant state progresses, ASP resistance may be present as well.

Why do fasting C3 and postprandial ASP production decrease with ROSI treatment? The putative function of ASP in adipose tissue is to promote storage of TG. There was a substantial lowering of ASP output from adipose tissue in response to ROSI, something that was clearly dissociated with minimal change in the transcapillary flux of fatty acids. There are multiple potential mechanisms to explain these changes. In principal, ASP could either play a minor role in this process, due to ASP resistance, or the action of ASP has been drastically changed by ROSI. Treatment with ROSI results in major changes in glucose homeostasis and increased insulin action (review [38]), thereby reducing the necessity of increased ASP. ROSI-mediated enhanced insulin action on adipose tissue may decrease postprandial ASP production, possibly because less ASP is required to exert the same function. At the level of adipose tissue, (i) decreased NEFA release due to effective postprandial suppression of hormone sensitive lipase action, (ii) increased lipoprotein lipase to enhance postprandial TG clearance, and (iii) increased intracellular capacity to channel NEFA for TG synthesis would all decrease the demands of ASP action/compensation. The changes in fatty acid transcapillary flux are an indirect reflection of all these processes, and the shift towards a more normal flux with ROSI treatment may indicate not only an improvement in insulin action on adipose tissue, but also an improvement on ASP action on adipose tissue. ROSI has been shown recently to have effects on all these processes, including increases in adipose tissue expression of FAT/CD36 and FABP4 (both important in NEFA channelling) and lipoprotein lipase [33]. Lastly, ASP and C3 may decrease because of up-regulation of the ASP receptor C5L2, increasing ASP sensitivity. We propose that, in a situation comparable to increased circulating insulin reflecting insulin resistance that increased plasma ASP is indicative of ASP resistance. By analogy, decreased postprandial ASP production suggests greater ASP sensitivity, with less ASP needed for the same action. In support of this, we have recently demonstrated that ROSI increases C5L2 expression and ASP functional response in adipocytes [39].

What is the mechanism both for the increased production of ASP in diabetic subjects and for the decreased ASP generation with ROSI production? ASP is produced through the cleavage of C3, via interaction with factor B and adipsin (all produced in adipocytes). In vitro in human adipocytes, both insulin and chylomicrons increase ASP through direct effects on increased C3 expression and secretion, and this may be one mechanism for the increased ASP (increased substrate availability) and C3 in diabetic subjects [26]. However, with ROSI treatment, changes in circulating postprandial C3 were relatively small, but postprandial generation of ASP was completely blocked. This is even more striking considering that only a small percentage of C3 is converted to ASP. Conversion of C3 to ASP is tightly regulated: in plasma, all three components required are freely circulating, yet little ASP is present. In vitro, mixing the three proteins together does not result in ASP generation, unless artificially activated [10]. It has been suggested that a specific cell surface component is required for conformational activation, although the nature of that site is unknown. In vivo in the present study, the adipose tissue bed is competent for ASP generation, but a trans-muscle gradient was negative. Therefore we hypothesize the existence of a controlled mechanism which initiates ASP generation, which is influenced by ROSI treatment.

## Conclusion

In summary, ASP production appears to be specific to the adipose tissue milieu, and is increased in diabetic subjects both fasting and postprandially. ROSI treatment, in addition to the many other demonstrated actions of this potent class of drugs, also decreases ASP production in adipose tissue.

## Abbreviations

The abbreviations used are: ASP: acylation stimulating protein; ANOVA: analysis of variance; A-V: arteriovenous; AUC: area under the curve; BMI: body mass index; NEFA: non-esterified fatty acid; ROSI: rosiglitazone; TG: triglyceride

## Authors' contributions

YT (Canada) carried out laboratory analysis, performed data analysis and drafted the manuscript. FK (United Kingdom) conceived of the study and participated in design and coordination. GT (United Kingdom) participated in design and coordination of the study, data collection and analysis. KC (Canada) participated in data analysis, statistical analysis and manuscript completion. All authors contributed to manuscript editing and all authors read and approved the final manuscript.
